# Intestinal perforation caused by fishbone in a child with the misdiagnosis of acute appendicitis: A case report

**DOI:** 10.1002/ccr3.4584

**Published:** 2021-08-06

**Authors:** Sang Ngoc Nguyen, Tuan Duy Nguyen, Lam Tung Vu, Cuong Ngoc Bao Hoang

**Affiliations:** ^1^ Haiphong University of Medicine and Pharmacy Haiphong Vietnam; ^2^ Haiphong Children's Hospital Haiphong Vietnam

**Keywords:** children, fishbone, intestinal perforation

## Abstract

If a child has abdominal pain, it is important to evaluate the possibility of intestinal perforation caused by foreign objects such as fishbone. If a foreign agent is present, laparoscopic surgery is an effective method to remove the foreign objects.

## Graphical abstract

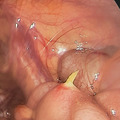

## 1 INTRODUCTION

Fishbone perforation is a severe complication that can cause peritonitis and damage to nearby organs. A case of fishbone perforation in a boy was misdiagnosed as appendicitis. The case we present is rare in children. A 5‐year‐old boy presented with right lower abdominal pain, mild fever, and constipation. He was diagnosed with acute appendicitis and was sent to the operating room for laparoscopic surgery. During surgery, the appendix was normal, and we discovered that a fishbone pierced the ileum wall. We removed that foreign object and sewed the intestine. He recovered well and was discharged after 7 days of treatment in stable condition. It is often difficult to diagnose intestinal perforation caused by a fishbone because the patient and family cannot remember the history of fishbone ingestion. Depending on the lesion location, children have different clinical characteristics, including constipation, abdominal pain, and anal pain. When a child has abdominal pain, a detailed history of eating and drinking, as well as a thorough physical examination and diagnostic imaging, is needed to make an accurate diagnosis.

Fishbone perforation has been described by several authors in the medical literature.^1^, ^2^, ^3^, ^4^ Perforation by fishbone is a dangerous condition that is seldom observed. Organ damage can result from intestinal perforation, particularly peritonitis, a severe medical condition that necessitates surgery. We present a case of fishbone perforation in a 5‐year‐old boy who was treated at Haiphong Children's Hospital, Vietnam.

## 2 CASE PRESENTATION

**FIGURE 1:**
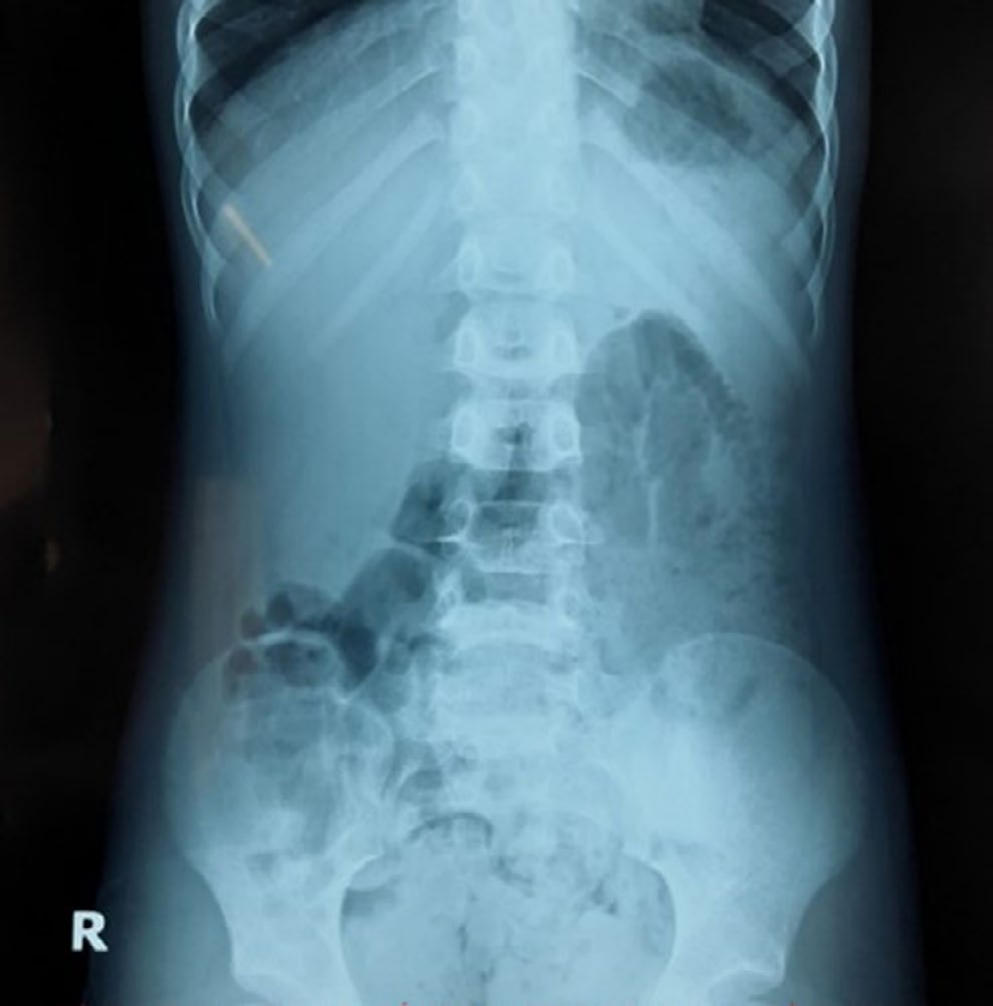
The patient's abdominal X‐ray

A 5‐year‐old boy with normal past medical history was admitted to Haiphong Children's Hospital with abdominal pain around the navel after having symptoms for 5 h. He also had a mild fever and an inability to pass stool or gas. On physical examination, the boy was conscious, able to communicate but tired, with a temperature of 37.5°C. His pulse rate was 100 beats per minute; his blood pressure was 90/60 mmHg; and the respiratory rate was 30 breaths per minute. His height was 100 cm, and he weighed 18 kg. All of these numbers were in the normal range for his age. The abdominal examination noted that he had abdominal distension and obvious abdominal muscle guarding in the right iliac and umbilical region. His complete blood count: the number of white blood cells was 14.8 × 10^9^/L, neutrophils accounted for 75.8%, and serum C‐reactive protein (CRP) was 21 mg/L. The abdominal ultrasound result showed that his intestines contained fluid and increase motility. The abdominal X‐ray did not show abnormal images (See Figure 1).

**FIGURE 2:**
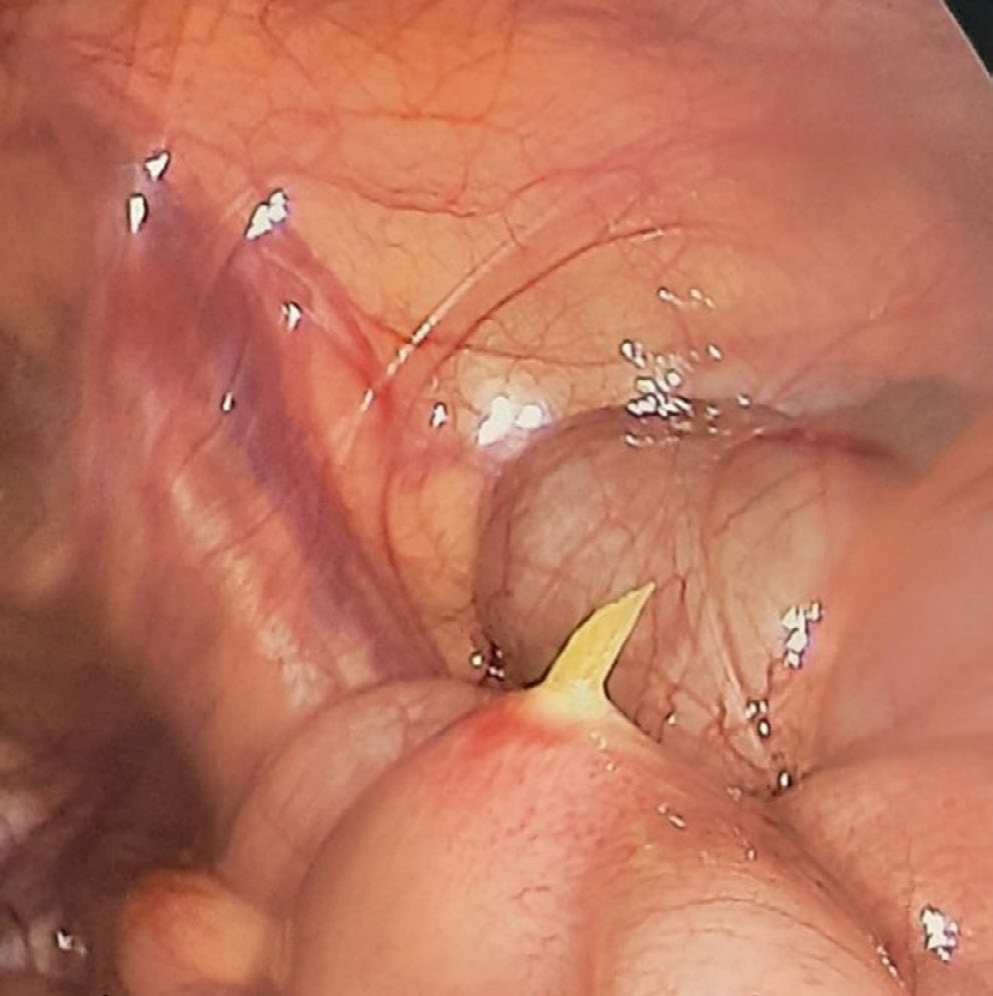
Fishbone punctured the intestine from the lumen of the intestine to the outside

**FIGURE 3:**
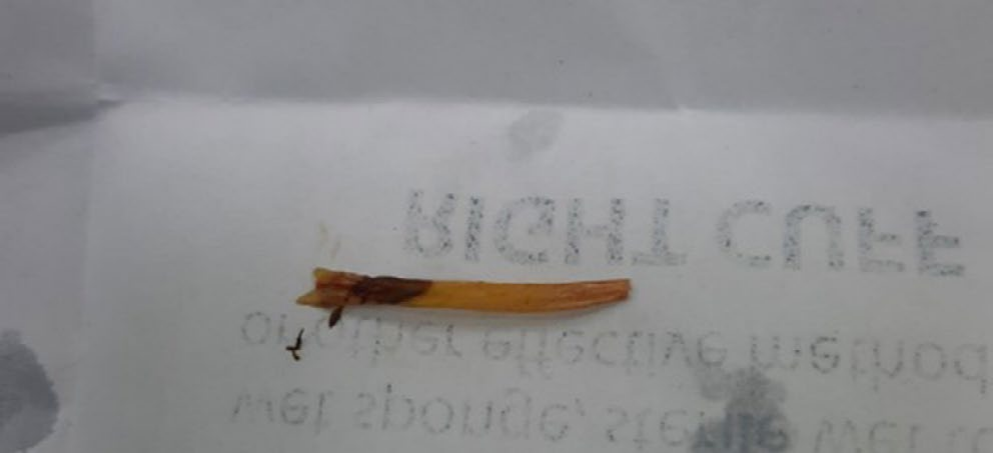
Full image of the fishbone

He was diagnosed with acute appendicitis and had had laparoscopic surgery. We saw little fluid around intestines through the laparoscopic camera, with no pseudomembrane, no Douglas fluid, and a normal appendix. While examining the ileum, we did not find Meckel's diverticulums but a sharp foreign object that punctured the intestine from the lumen of the intestine to the outside, about 35 cm from the ileum—cecum (See Figures 2 and 3). After that, we removed the foreign object, stitched the hole in the intestine, cleaned the abdomen, and closed incisions.

The boy made a full recovery and was discharged in a satisfactory condition following 7 days of treatment

## 3 DISCUSSION

Fishbone perforation is rarely seen, especially in children. Although most foreign objects can be excreted within a week of entering the gastrointestinal tract,^1^, ^5^ 1% of patients may still suffer from intestinal perforation if the fishbones were long or sharp. Moreover, it is complicated to make a precise diagnosis because patients or their families cannot make sure what foreign object they ate in their meals. So, these cases are often misdiagnosed as acute appendicitis.

Depending on the perforation's location, patients may have various clinical symptoms, including constipation, abdominal pain, and anal pain. A study reported that 95% of patients presented with abdominal pain, which is the most crucial symptom, 81% having a fever, and 39% having local peritonitis.^2^ In most cases of intestinal perforation caused by a foreign object, the foreign agent is a fishbone with a pointed shape. In some countries or regions that prefer eating fish, gastric perforation or other complications caused by fishbone are very common.^6^ While fish bones may induce intestinal perforation in any section of the intestine, it is more commonly seen in areas of physiological stenosis or intestinal transitions such as the ileum or rectosigmoid junction.^7^ In a study, the probability of ileal perforation was 83%.^8^ In another report, the perforation of the end of the ileum was 38.6%, but the jejunum rate was lower, just 14.3%.^5^

Patients do not often think they could suffer from intestine perforation due to a fishbone. Therefore, it is hard for doctors to take a medical history of the disease. The fishbone is often only found during an imaging examination or surgical exploration.^6^ Imaging examination is usually unreliable in the diagnosis of these cases. High‐density shadow, free gas, and abscess formation are often used to determine the presence of inflammatory changes or perforation.^9^ However, the fishbone gradually penetrates the intestinal wall through extrusion. The perforation site is often covered by fibrin or adjacent intestines, limiting the outflow of intestinal contents and reducing the possibility of the free gas appearance in abdominal X‐ray films.^10^ Free intestinal gas found in X‐ray films accounted for only 20% of patients.^8^ In another study with 358 patients suffering from fishbone perforation, X‐rays were only 32% sensitive.^3^ The fishbone is also affected by radiation doses, inflammatory tissues, or liquids around the damage.^4^

This child's most essential symptoms were abdominal pain, clear abdominal muscle guarding in the right iliac region with constipation, and fever in our patient. Diagnostic imaging results were not sensitive. Therefore, this patient's symptoms are somewhat close to those of a patient with acute appendicitis, perhaps leading to a misdiagnosis of abdominal pain caused by acute appendicitis.

Gastroscopy and enteroscopy are popular methods to remove the gastrointestinal foreign bodies. Only 1% of cases need surgical excision. However, depending on the perforation site and clinical symptoms, treatment could be chosen through suture perforation site, bowel resection, or Hartman procedure.^8^ Laparoscopic surgery caused less damage than traditional laparoscopic surgery, so it has gradually replaced the traditional method of abdominal open exploration. Currently, laparoscopic surgery is the preferred method of choice.^11^ In our case, because there was no precise diagnosis, we chose laparoscopic surgery. During the surgery, we found a sharp fishbone punctured the intestine from the lumen of the intestine to the outside, about 35 cm from the ileum—cecum, and the patient's appendix was normal. We removed the fishbone, stitched the hole, cleaned the abdomen, and closed the incisions.

The boy recovered very well and was discharged after 7 days of treatment in stable condition. Nevertheless, after all of this, his family still cannot remember clearly that whether their son ate fish that day or not. All they can make sure that their family regularly ate fish for dinner.

## 4 CONCLUSIONS

Identifying a case of fishbone ingestion is difficult because patients often do not remember when they ate fish, especially children. Therefore, it is critical for parents to recall their children's food and drinking history. In the case of a child with acute abdomen, doctors have to ask carefully about eating and drinking histories, if they ate fish, chicken, and so on, or not. A clinical examination together with imaging diagnosis is necessary to make the most accurate diagnosis. When performing an inflammatory appendectomy, the abdomen should also be carefully examined.

## 5 RECOMMENDATIONS

When dealing with a child having an abdominal pain, it is important to evaluate the possibility of intestinal perforation caused by a foreign object such as a fishbone, in addition to appendicitis. If a foreign agent is present, laparoscopic surgery to remove the foreign objects and suture the gastrointestinal perforation is a common and effective method.

Families should not feed their children with fish that still has its bones inside. Parents need to remove all the bones from the fish before their child consumes it.

## CONFLICT OF INTEREST

The authors declare that they have no competing interests.

## AUTHOR CONTRIBUTIONS

SNN, TDN, LTV, and CNBH participated in the study design, protocol development, and performance, data analysis, interpretation of data, writing the manuscript, carrying out the clinical data collection and data analysis, and observing the patient during the treatment. TDN and CNBH performed laparoscopic surgery. All authors read and approved the final manuscript.

## ETHICAL APPROVAL

Approval for the study was obtained from Medical Ethics Council of Haiphong University of Medicine and Pharmacy, and informed consent was obtained according to the Declaration of Helsinki.

## PATIENT CONSENT

Written informed consent was obtained from the patient and his parent for publication of these data and the accompanying images. A copy of the written consent is available for review by the Editor of this journal.

## Data Availability

The data that support the findings of this study are available from the corresponding author upon reasonable request.
